# Validity and Internal Consistency of a Rubric for Cervical Collar Placement in Nursing Students

**DOI:** 10.3390/nursrep16070250

**Published:** 2026-07-17

**Authors:** Jose Miguel Diez-Fernandez, Tania Fernandez-Villa, Amaia Rodriguez-Badiola, Elba Mauriz, Carmen Crespo-Martinez, Ana Vazquez-Casares

**Affiliations:** 1 Department of Nursing and Physiotherapy, University of León, 24071 Leon, Spain; jdiezf@unileon.es (J.M.D.-F.); arodrb16@estudiantes.unileon.es (A.R.-B.); elba.mauriz@unileon.es (E.M.); mcrem@unileon.es (C.C.-M.); 2 Research Group on Gene-Environment Interactions and Health (GIIGAS), Institute of Biomedicine (IBIOMED), University of León, 24071 Leon, Spain; tferv@unileon.es; 3 Biomedical Research Network Centre for Epidemiology and Public Health (CIBERESP), 28029 Madrid, Spain

**Keywords:** rubric, cervical collar, internal consistency, reliability, nursing, students

## Abstract

**Background/Objectives:** The assessment of clinical competencies in nursing education requires valid and reliable instruments, especially for essential procedures such as the placement of a cervical collar in the care of polytrauma patients. The objective of this study was to analyse the psychometric properties of a rubric designed to assess the placement of cervical collars in nursing students in a low-fidelity clinical simulation environment. **Methods:** A quasi-experimental, cross-sectional study was conducted with 186 undergraduate nursing students, organised into 61 groups, over three academic years (2021–2024). An eight-item rubric was applied, with a four-level Likert scale (1–4). Interrater reliability was analysed using Cohen’s Kappa index (0.418–0.796), internal consistency using Cronbach’s alpha (alpha = 0.753), and the internal structure of the instrument using exploratory factor analysis, applying Varimax orthogonal rotation. **Results:** Inter-rater reliability showed values ranging from moderate to substantial, with greater agreement observed in items related to the selection of the collar and the position of the medical team, and moderate agreement in those related to technical manoeuvres and immobilisation. The rubric showed adequate overall internal consistency (α = 0.76), with good to very good consistency values in six of the eight items. Exploratory factor analysis identified a two-dimensional structure with a dominant procedural factor (four items) and a second factor associated with clinical judgement (four items). **Conclusions:** The validated rubric has adequate levels of reliability and internal consistency for evaluating the placement of cervical collars by nursing students. Its application can promote more objective and structured evaluation processes in clinical simulation for nursing students, contributing to the development of essential skills in the care of polytrauma patients.

## 1. Introduction

Nursing education has undergone significant changes in recent decades, gradually shifting from models focused on the transmission of theoretical content to educational approaches geared toward the integrated development of knowledge, technical skills, and complex clinical competencies. This change responds to the need to prepare future professionals for increasingly demanding healthcare environments, where decision-making, patient safety, and clinical reasoning play a fundamental role [1,2].

In this context, clinical simulation has established itself as an effective pedagogical strategy in healthcare training, allowing for the training of technical and non-technical skills in controlled and safe environments. Several studies have shown that simulation allows for the integration of theory and practice, promotes the progressive acquisition of skills, improves clinical performance, and increases the safety of real patients, both for students and professionals in continuing education [3,4,5,6,7].

Among the essential manoeuvres in the initial care of polytrauma patients is the placement of a cervical collar, a key procedure for preventing secondary spinal cord injuries and ensuring adequate stabilisation of the cervical spine. Despite being a widely protocolized technique, the literature describes considerable variability in its execution, especially in training contexts, which can compromise clinical safety and quality of care [8,9]. This variability highlights the importance of having not only clear protocols but also assessment tools that allow for objective evaluation of the level of competence achieved.

In education, rubric-based assessment has been proposed as a structured and transparent strategy for evaluating clinical performance, breaking down complex procedures into observable criteria and establishing progressive levels of mastery. Rubrics contribute to improving the objectivity of assessment, facilitate formative feedback, and promote self-regulation of learning, especially when self-assessment and peer assessment processes are integrated [10,11,12]. However, despite the existence of well-defined technical protocols for cervical collar placement, the literature shows a shortage of validated instruments specifically designed to assess this skill in clinical simulation settings, particularly in the context of nursing education. This lack of psychometrically sound tools limits the standardisation of assessment and makes it difficult to compare results across training cohorts and teaching contexts [3,7,13,14].

Within this framework, the objective of the present study was to analyse the psychometric properties—interrater reliability, internal consistency, and factorial structure—of a rubric designed to assess the technique of cervical collar placement in nursing students in a clinical simulation context. 

## 2. Materials and Methods

### 2.1. Design

A quasi-experimental, cross-sectional study was conducted to validate a rubric designed to assess the technique of placing a cervical collar in a clinical simulation setting. This study was conducted at a public university in Spain as part of a compulsory subject in the Nursing Degree programme. The cross-sectional design allowed for a snapshot of the performance of participants in different academic cohorts and an assessment of the applicability, consistency, and relevance of the rubric. This study considered four fundamental aspects: (1) content validity (assessed by experts); (2) inter-rater reliability (determined using the Kappa index); (3) internal consistency (estimated using Cronbach’s alpha coefficient); and (4) internal structure using factorial analysis.

The data were collected in February of 2022, 2023, and 2024 in five 2 h sessions each academic year.

### 2.2. Population and Sample

The sample consisted of nursing degree students enrolled in the course ‘Fundamentals of Health Care, Special Care’, corresponding to the second year of the curriculum, in the academic years 2021–2022, 2022–2023 and 2023–2024. Convenience sampling was used, with a total of 186 students divided into 62 groups of 3 people each. Participation was voluntary and anonymous, and verbal informed consent was obtained from the students. The sample size was determined using the QuestionPRO sample size calculator [15], considering a 95% confidence level, a 5% margin of error and an expected proportion of 50%, which yielded a minimum requirement of 170 participants.

### 2.3. Instruments

An ad hoc rubric was used to evaluate the cervical collar placement procedure. This tool was developed based on the judgement of three experts with clinical experience and teaching experience in emergency medicine and clinical simulation. Prior to each academic year, the three instructors participated in a joint calibration session in which the rubric criteria, performance levels and expectations of the procedure were reviewed and agreed upon, thus guaranteeing continuity and reliability between evaluators.

The rubric used consists of eight items covering the steps of selecting, measuring, and placing the collar. Each item is scored on an ordinal rating scale from 1 to 4, where 1 represents incorrect or poor performance, and 4 represents autonomous and correct performance. The maximum score is 32 points and the minimum is 8 points. This structure allows for the assessment of both technical and behavioural aspects of clinical performance in simulation.

To reduce information bias, the scale administration procedure was standardised. It was delivered in paper format to each group before the procedure was performed, using uniform instructions and the supervision of three trained instructors, who were responsible for all administrations to ensure the homogeneity of the process over the three years. The items were scored by the students themselves, according to the levels of mastery established in the rubric, which allowed for the application of uniform criteria and reduced variability in the interpretation of the items, minimising the risk of evaluator bias. Group scoring was carried out by consensus among the members of each group prior to recording the final score. No individual scoring system or tie-breaking mechanism was established, as the assessment was constructed collaboratively. No group reported disagreements regarding this scoring procedure during the study.

### 2.4. Procedure

The intervention was carried out in a clinical simulation classroom during a two-hour practical session, in which a scenario involving the fitting of a cervical collar was developed. In order to include all enrolled students, the session was repeated four times in each academic year, over a period of three years.

Each session involved five teams made up of two groups of three students each, encouraging active participation and collaborative learning.

**Figure 1:**
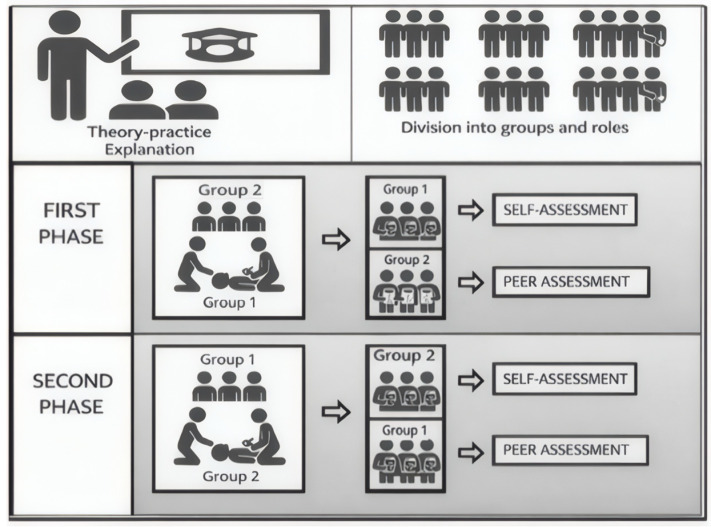
Simulation scenario development procedure based on evaluation methods.

- Start (Briefing): The instructor presented the task to be performed—fitting a cervical collar—and gave a brief theoretical explanation of the technique, followed by a practical demonstration and presentation of the assessment rubric, including its structure, the levels of proficiency it comprises, and the criteria for assigning scores. The students organised themselves into groups of three, taking on the roles of victim, responder 1 and responder 2. Each group was assigned a number and paired with another group to carry out the activity.
- Development of the scenario: The students placed the cervical collar under the direct supervision of the instructor, ensuring uniform application of the rubric and promoting adherence to clinical practice standards. The process was carried out in two phases determined by the mode of assessment of the scenario used: peer assessment or self-assessment (Figure 1).
  - First phase: Group 1 performed the procedure and Group 2 observed. Upon completion, Group 2 evaluated Group 1 (peer evaluation), while Group 1 performed its self-evaluation.
  - Second phase: Group 2 performed the procedure and Group 1 observed. Upon completion, Group 1 evaluated Group 2 (peer evaluation), while Group 2 performed its self-evaluation.

The data were analysed using IBM SPSS Statistics version 26.0 (IBM Corp., Armonk, NY, USA). A cleaning and verification process was performed beforehand to identify missing values, duplicates, and inconsistencies.

### 2.5. Variables

The independent variables are the group, the academic year, and the assessment method (self-assessment or peer assessment). The dependent variables correspond to the mastery indicators and performance levels of the rubric items.

### 2.6. Analysis

Measures of central tendency and dispersion (mean, mode, standard deviation) were obtained for the dependent variables, and a one-factor ANOVA was performed to analyse the differences between groups. A statistical significance level of *p* < 0.05 was established.

The normality of the dependent variables (items) was analysed using the K-S (Kolmogorov–Smirnov) test (*p* = 0.001) and the homogeneity of variances using Levene’s test (*p* = 0.001). Although the Kolmogorov–Smirnov and Levene tests indicated non-normality and heterogeneity of variances (*p* = 0.001), ANOVA was considered appropriate given the robustness of this test against moderate violations of normality assumptions with sufficiently large sample sizes (*n* = 186). Additionally, the Kruskal–Wallis test was applied as a non-parametric alternative, yielding consistent results.

Although no predefined confounding variables were included, the academic year was considered a potential influencing factor and was analysed specifically to minimise a possible cohort effect. In accordance with STROBE guidelines, analytical subgroups were explored, comparing scores between the three academic years (2021–2022, 2022–2023, 2023–2024) and between the two assessment methods. Likewise, the possible interaction between course and method was examined exploratorily by comparing means in each combination using descriptive analyses and ANOVA.

Cohen’s Kappa index was calculated for each item by comparing the scores assigned through self-assessment by the performing group with those awarded through peer assessment by the observing group, in order to determine the degree of agreement between both assessment methods. It should be noted that this comparison does not equate to inter-rater reliability in the strict sense, as the two records do not come from independent trained raters but from groups with different roles within the simulation scenario. Kappa values were interpreted according to the classification proposed by Landis and Koch (1977): values < 0 indicate agreement worse than chance; 0.00–0.20 slight agreement; 0.21–0.40 acceptable agreement; 0.41–0.60 moderate agreement; 0.61–0.80 substantial agreement; and 0.81–1.00 almost perfect agreement [16].

The internal consistency of the instrument was analysed using Cronbach’s alpha coefficient, both for the rubric as a whole and for the different subgroups of items that comprise it, with the aim of evaluating the degree of internal homogeneity and the correlation between items, as well as their contribution to the common construct. Cronbach’s alpha assumes that the items measure the same dimension and that the responses have a logical order. The interpretation of Cronbach’s alpha values was carried out following the classification proposed by Tavakol and Dennick [17], which is widely accepted in the fields of health sciences and education. According to this classification, values of α ≥ 0.90 indicate excellent consistency; between 0.80 and 0.89, very good; between 0.70 and 0.79, good; between 0.60 and 0.69, moderate; between 0.50 and 0.59, low; and values below 0.50, very low.

The internal structure of the rubric and its structural validity were assessed using exploratory factor analysis (EFA). An EFA was selected over confirmatory factor analysis (CFA) since there is no previous validated structural model specifically for the evaluation of cervical collar placement in nursing. The EFA allows us to inductively identify the dimensions underlying the items of the rubric without imposing a predefined factor structure, which is especially suitable in the initial phases of development and validation of competency assessment instruments and to explore whether the items are grouped around the theoretically expected dimensions—technical-procedural execution and clinical judgement. Although self-assessment and peer-assessment records were treated as independent observations in order to maximise the available sample size, we acknowledge that intragroup correlation may exist given that both records derive from the same scenario. However, since they represent distinct evaluative perspectives, we consider this approach justified as an exploratory approximation in this initial validation phase. Future studies should consider the use of multilevel models to explicitly account for this dependency.

The suitability of the data for exploratory factor analysis was evaluated using the Kaiser–Meyer–Olkin (KMO) sample adequacy index and Bartlett’s sphericity test. KMO values ≥ 0.60 were considered acceptable, while a statistically significant Bartlett test (*p* < 0.05) was interpreted as indicative of sufficient correlations between items for the application of factor analysis. Factor retention was based on the criterion of eigenvalues greater than 1 (Kaiser criterion) and on inspection of the scree plot. A factor solution that explained at least 50% of the total variance was considered adequate. To facilitate the interpretation of the factors, a Varimax orthogonal rotation with Kaiser normalisation was applied, with the aim of maximising the variance explained by each factor. For the interpretation of the factorial structure, a minimum factor loading 0.40 in a single factor was established as the criterion for retaining items. Items with loadings below this value or with significant cross-saturations were reviewed for possible elimination or conceptual reassignment. Although self-assessment and peer-assessment records were treated as independent observations in the exploratory factor analysis to maximise the available sample size, we acknowledge the potential intragroup correlation as a limitation of this approach.

### 2.7. Ethical Considerations:

This study was conducted in accordance with the principles of the Declaration of Helsinki and current regulations on educational research. Student participation was voluntary, the objectives of the study were explained in advance, and informed consent was obtained. The confidentiality of the information and the impossibility of identifying individual participants were always guaranteed.

## 3. Results

The experts reviewed and evaluated the initial tool in terms of clarity, relevance, and appropriateness to ensure the validity of the content before its use. The initial tool included 10 domain indicators as performance indicators, following recognised standards and clinical guidelines. Following their evaluation, the experts proposed a final tool that eliminated two items related to device selection, in line with the lack of implementation of these items in the proposed scenario and improved the wording of two of the eight remaining items. Two items included in the initial version of the rubric—relating to the assessment of the need for cervical immobilisation—were deleted after evaluation by the experts. Their exclusion is due to the fact that the students did not have decision-making capacity in these items; therefore, they could not be evaluated. These items could be reintegrated and validated in higher-fidelity simulation environments, in which they constitute a competence to be assessed. Thus, the final rubric used consists of eight domain indicators and four levels of performance or achievement (Table 1).

In the 2021–2022 academic year, 21 teams were completed; in 2022–2023, 16 teams were completed; and in 2023–2024, 25 teams were completed. The total number of students participating was 186, organised into 62 groups. One record was removed from a team that did not complete the scoring of all items (either in self-assessment or peer assessment) for the 2022/2023 academic year. A review of this case confirmed that the missing data were not associated with any systematic pattern related to prior performance, group membership, or academic year, and therefore did not introduce systematic bias into the analysis. Thus, the sample consisted of 183 participants in 61 groups.

The measures of central tendency and dispersion obtained by the groups in the rubric are shown in Table 2, according to the mode of assessment (self-assessment and peer assessment) and the academic year of the participants.

The scores obtained through the rubric by the assessment and peer assessment methods show no significant differences either in the assessments of the 61 groups in total (*p* = 0.34) or by academic year: (2021–2022: *p* = 0.25; 2022–2023: *p* = 0.33; 2023–2024: *p* = 0.45). A comparison of the scores obtained in each assessment method in each academic year shows that the 2021–2022 academic year has the lowest scores in the rubrics for both self-assessment and peer assessment.

In relation to the score obtained in the rubric using each assessment method in each academic year, significant differences are detected in the values obtained through self-assessment (*p* < 0.001) and peer assessment (*p* < 0.001), indicating variability in performance according to the academic cohort, with lower scores in the 2021–2022 academic year.

The frequency distribution of the scores (domain measures: 1, 2, 3 and 4) given by students through the two assessment methods (self-assessment and peer assessment) for each of the items in the rubric is shown in Table 3.

There is a tendency to give items higher scores (levels 3 and 4), especially in items 1 (collar selection), 4 (position of medical equipment and instructions) and 5 (neck measurement). In the distribution of frequencies attributed to each item in peer assessment, a greater dispersion of scores was observed, with values at lower levels in items 2, 3, 6, 7 and 8.

### 3.1. Inter-Rater Reliability

Table 4 shows the Kappa index values obtained for each item in the rubric, calculated from the comparison between the score assigned by the performer themselves and that awarded by their peers.

Substantial agreement was observed in three of the eight items evaluated (items 1, 4, and 6), indicating a high degree of concordance among evaluators. The remaining five items (items 2, 3, 5, 7, and 8) showed moderate agreement, reflecting adequate concordance. No item had an agreement lower than 0.40 or higher than 0.80, which shows consistent and acceptable inter-rater reliability for the rubric used.

### 3.2. Internal Consistency

The total Cronbach’s alpha obtained for the rubric is 0.753, indicating good internal consistency. Table 5 shows the Cronbach’s alpha value for each item, as well as the associated degree of consistency, allowing for analysis of the individual contribution of each element of the instrument.

Of the eight items in the rubric, two showed moderate consistency (items 3 and 7), two achieved acceptable consistency (items 5 and 6), three showed very good consistency (items 1, 2, and 8), and one item showed excellent consistency (item 4). These results indicate that most of the items in the rubric contribute strongly to the overall construct, reinforcing the internal reliability of the instrument.

### 3.3. Factor Analysis

Given that the rubric assesses the same competency through the same items, the EFA was performed jointly on the set of assessments, considering each record as an independent observation of the type of assessment performed. Table 6 shows the matrix of components of the domain indicators that make up the rubric.

Exploratory factor analysis identified a two-factor structure with eigenvalues greater than 1, which together explained 55.7% of the total variance.

Factor 1 explained 37.7% of the variance and grouped items related to the positioning and immobilisation of the victim (items 3, 4, 7, and 8), suggesting a procedural dimension.

Factor 2 explained 18.0% of the variance and grouped items related to the selection, handling, measurement, and placement of the collar (items 1, 2, 5, and 6), forming a distinct dimension with a greater component of clinical judgement and decision-making.

The resulting structure showed a balanced distribution of items between both factors, with four items in each dimension, reinforcing the internal consistency and applicability of the rubric in the evaluation of the procedure.

The rotated component matrix (Varimax) showed factor loadings between 0.42 and 0.85 in the first factor and between 0.48 and 0.79 in the second. Item 8 showed slight cross-saturation, with a lower secondary loading in the second factor, so it was kept in the procedural dimension in view of the conceptual and clinical consistency of the instrument.

## 4. Discussion

The aim of this study was to analyse the psychometric performance of a rubric designed to assess the placement of cervical collars in nursing students. The results obtained allow us to interpret the variability observed between academic cohorts, as well as to assess the inter-rater reliability, internal consistency and factorial structure of the instrument in a clinical simulation context.

One of the most relevant findings was the greater intragroup variability observed in the 2022–2023 academic year, both in self-assessment and peer assessment. Given that the assignment of students to groups, the timing of the activity, and the teaching conditions were homogeneous in the three courses, this variability does not appear to be attributable to organisational factors. In the same sense, the lower scores recorded in the 2021–2022 academic year, despite identical teaching conditions, could respond to the natural heterogeneity between groups in the levels of basic competence and in previous learning trajectories, rather than to an educational limitation of a systemic nature. These patterns suggest that the observed differences may be related to the overall performance of the group or the degree of previous acquisition of competences, which highlights the ability of rubrics to detect differences between training groups and reinforces their discriminatory value as an assessment tool in higher education [18].

In relation to the distribution of scores per item, a general trend towards high performance levels was observed in those items associated with more objectifiable and easily observable actions. Conversely, items involving greater technical precision, coordination, or postural control showed a greater dispersion of scores. This pattern is consistent with the greater technical and cognitive complexity of certain clinical manoeuvres and has been previously described in the assessment of competencies using rubrics in healthcare contexts [7,14,19].

From the point of view of inter-rater reliability, the Kappa index values indicated moderate to substantial levels of agreement for most items. The lowest values were concentrated in those items with more interpretative descriptors, especially those related to the neutral position of the neck and bimanual immobilisation. The absence of objective or quantifiable criteria and the lack of discrimination between certain levels of performance may favour heterogeneous interpretations among assessors, a phenomenon widely described in the literature on clinical assessment with rubrics [11,14,18,19]. The inter-rater variability observed in this study is consistent with that described in other training contexts, where discrepancies in the interpretation of procedural items by different assessors are common, especially when they involve interpretative components. This phenomenon is characteristic of standardised clinical procedures, such as those included in teaching guides on the placement of cervical collars, including the institutional guide of the University of Murcia [20].

However, this degree of variability is common in instruments used for training purposes, where assessment is conceived as a dynamic process that includes feedback and evaluative dialogue. In this sense, moderate levels of inter-rater reliability do not necessarily compromise the pedagogical usefulness of the instrument when structured feedback processes are integrated [21,22].

The internal consistency analysis showed an adequate overall Cronbach’s alpha value. In the field of health sciences education, alpha values around 0.70–0.80 are considered acceptable when assessing complex clinical skills that integrate technical, cognitive, and procedural components [17,23]. The items with the highest interpretative load, specifically items 2, 3, 5, and 6, showed lower reliability values compared to the more technical and observable items. This pattern has been described in recent studies on the validation of clinical instruments, where elements that require greater evaluative judgement tend to show lower inter-rater agreement [7,13,14,19]. In particular, the moderate consistency observed in items 3 (neutral neck position) and 7 (bimanual immobilisation) may be attributed to the greater subjectivity inherent in their descriptors, as both involve judgements about body positioning and motor coordination that are more difficult to objectify independently of the observer. Future versions of the rubric should consider revising and refining the descriptors of these items to reduce inter-rater variability. Furthermore, in procedural skill assessment tools, a moderate alpha value should be interpreted as reflecting the independence of each item rather than a limitation, given that each step of the procedure contributes unique and non-redundant information to the overall evaluation of competence, which is considered a desirable property of formative assessment instruments.

From a structural perspective, the exploratory factor analysis performed jointly provided additional evidence of the internal validity of the rubric by identifying a two-dimensional structure consistent with its theoretical framework. A first factor mainly grouped items related to the technical-procedural execution of cervical collar placement, while a second factor integrated items with a greater interpretative load, associated with the measurement and correct adjustment of the device. This differentiation reflects the coexistence of technical components and clinical judgement in performance assessment, as described in recent clinical assessment instruments [7,23].

Likewise, the presence of some items with moderate loadings on both factors suggests a mixed nature typical of complex clinical procedures, in which technical execution and clinical assessment are closely interrelated. Overall, these results reinforce the competency-based approach of the rubric and support its usefulness in training contexts, in line with studies that indicate that reliability does not need to be maximum to fulfil an effective educational function when well-designed instruments are used and accompanied by structured feedback [24,25].

Limitations and future directions:

One of the main limitations of this study is that the sample consisted exclusively of undergraduate nursing students, which may restrict the generalisation of the results to other groups of health science students and professionals. Given that the placement of a cervical collar is a technique shared by different healthcare professionals, it would be advisable to extend the validation of the instrument to other degrees with responsibility for this procedure, as well as to different educational and training levels.

Among the limitations of this study, it should be noted that, although the panel of experts evaluated the items in terms of clarity, relevance and adequacy, the Content Validity Index (CVI) was not calculated, which would have provided more rigorous quantitative evidence of the content validation process. It is recommended that this metric be incorporated into future reviews of the instrument.

Item 8, which presented a slight cross-saturation between both factors (0.42 and 0.32, respectively), reflects the dual nature of the verification step, which integrates both a technical and an interpersonal dimension. While the item remains in the procedural factor due to its primary factor load and clinical consistency, future research could explore whether subdividing this item into two separate indicators would improve the psychometric accuracy and discriminant validity of the instrument.

On the other hand, the use of low-fidelity mannequins in a controlled classroom environment, as a first-level scenario, may have limited the validity of the evaluation, since factors of real clinical practice were not contemplated, so the validation of the rubric in contexts of higher fidelity is recommended as a line of future research.

Furthermore, future studies could explore the application of the rubric in other teaching contexts and simulation scenarios, as well as analyse the impact of possible adjustments to the item descriptors on their psychometric performance, in order to optimise the reliability and validity of the instrument.

## 5. Conclusions

The proposed rubric has demonstrated adequate psychometric properties in terms of item analysis, showing an acceptable level of agreement and overall internal consistency, indicating that the rubric has a solid and homogeneous structure. The results of this study show that the rubric can be considered a reliable and consistent tool for assessing the technique of placing cervical collars in nursing students.

The agreement between assessors was adequate for most items, which supports its use in contexts involving self-assessment and peer assessment.

Furthermore, the internal structure identified in two factors confirms that the tool integrates both procedural skills and elements of clinical judgement, which are essential components in the development of advanced clinical competencies.

In the field of health sciences education, this rubric provides a structured framework that has the potential to improve the quality of feedback and promote more objective and standardised assessment processes in simulation-based training. Its application may, in the long term, favour the acquisition of clinical competencies relevant to the care of polytrauma patients. In this regard, formal integration of this instrument into nursing curricula is recommended as part of simulation-based clinical training programmes, given its potential to reduce variability in a high-risk clinical procedure and contribute to the standardisation of clinical practice.

Future longitudinal studies should empirically evaluate the impact of its use on patient safety outcomes.

## Figures and Tables

**Table 1:** Rubric for cervical collar application.

Domain Indicator	Levels of Achievement/Performance
1. Selection of the appropriate cervical collar	1. Does not select the cervical collar correctly rarely or never
	2. Selects the cervical collar correctly on most occasions
	3. Selects the cervical collar correctly almost always
	4. Always selects the cervical collar correctly
2. Handling (manipulation) of the cervical collar	1. Does not handle the cervical collar independently
	2. Handles the cervical collar independently only by following instruction or by imitation
	3. Handles the cervical collar independently on most occasions
	4. Always handles the cervical collar independently
3. Positioning of the victim and maintenance of the neck in a neutral position	1. Does not position the victim correctly and does not maintain the neck in the correct position.
	2. Positions the victim correctly and maintains the neck in the correct position but requires guidance for both actions.
	3. Positions the victim correctly, although some guidance is required, but maintains the neck in the correct position independently.
	4. Positions the victim correctly and maintains the neck in the correct position independently.
4. Appropriate positioning of healthcare per-sonnel	1. Neither the practitioner nor the rest of the team adopts an appropriate position
	2. Adopts an appropriate position but does not provide instructions to the rest of the team, or the instructions are incorrect
	3. Adopts an appropriate position and provides appropriate instructions to the rest of the team in almost all circumstances
	4. Adopts an appropriate position and provides appropriate instructions to the rest of the team in all circumstances
5. Neck measurement	1. Does not complete the measurement correctly
	2. Completes the measurement correctly by imitation
	3. Completes the measurement correctly but requires some guidance
	4. Completes the measurement correctly and interprets it appropriately
6. Correct application of the cervical collar	1. Does not apply the cervical collar correctly
	2. Applies the cervical collar correctly by imitation
	3. Applies the cervical collar correctly but requires some guidance
	4. Applies the cervical collar correctly independently
7. Bimanual immobilisation	1. Does not maintain bimanual immobilisation.
	2. Maintains bimanual immobilisation correctly by imitation or only partially.
	3. Maintains bimanual immobilisation correctly but requires some guidance.
	4. Maintains bimanual immobilisation correctly independently.
8. Verification of correct collar placement and victim comfort	1. Does not check correct placement, although attempts to ensure victim comfort, or neither checks placement nor ensures comfort
	2. Checks collar placement and correct it when necessary on some occasions, but does not ensure victim comfort, or does not check correct placement but does seek to ensure comfort
	3. Checks collar placement and correct it when necessary, but does not ensure victim comfort, or checks correct placement and comfort most of the time.
	4. Checks collar placement, corrects it when necessary, and always ensures victim comfort

**Table 2:** Descriptive analysis: measures of central tendency. Statistical significance of self-assessment and peer-assessment scores for the total sample and for each academic year.

Participants (n Groups)	Assessment Method	X (DE)	Mediana (P25–P75)	Min/Max	*p* Value
Total (61)	Self-assessment	27.69 (3.06)	29 (25–31)	18/32	0.34
	Peer assessment	27.43 (3.57)	28 (26–31)	18/32	
Academic Year 2021–2022 (21)	Self-assessment	24.29 (2.39)	24 (23–26)	20/29	0.25
	Peer assessment	24.05 (3.02)	23 (22–26)	18/32	
Academic Year 2022–2023 (15)	Self-assessment	29.87 (2.50)	31 (28–32)	18/32	0.33
	Peer assessment	29.00 (2.39)	30 (27–31)	25/32	
Academic Year 2023–2024 (25)	Self-assessment	29.24 (2.90)	30 (29–31)	20/32	0.45
	Peer assessment	29.32 (2.37)	30 (28–31)	21/32	

**Table 3:** Frequency distribution of scores in self-assessment and peer assessment.

Participants (n Groups)	Self-Assessment				Peer-Assessment			
	1	2	3	4	4	1	2	3
1: Selection of the appropriate cervical collar	0	1	8	52	0	2	10	49
2: Handling (manipulation) of the cervical collar	0	10	16	35	2	6	20	33
3: Positioning of the neck in a neutral position	3	12	16	30	3	7	20	31
4:Appropriate positioning of healthcare personnel	0	9	12	40	0	9	8	44
5: Neck measurement	0	5	8	48	0	6	13	42
6: Correct application of the cervical collar	0	11	19	31	1	10	25	25
7: Bimanual immobilisation	0	12	16	33	2	10	16	33
8: Verification of correct placement and comfort	0	10	15	36	3	5	17	36

Note: Cell background colors represent a heat map gradient based on frequency values, ranging from red (lowest frequency) to green (highest frequency).

**Table 4:** Kappa index: inter-rater reliability assessment and interpretation (Landis and Koch).

Item Assessed (Domain Indicator)	Kappa Index	Level of Reliability
1: Selection of the appropriate cervical collar	0.706	Substantial
2: Handling (manipulation) of the cervical collar	0.579	Moderate
3: Positioning of the neck in a neutral position	0.467	Moderate
4: Appropriate positioning of healthcare personnel	0.712	Substantial
5: Neck measurement	0.499	Moderate
6: Correct application of the cervical collar	0.796	Substantial
7: Bimanual immobilisation	0.418	Moderate
8: Verification of correct placement and comfort	0.520	Moderate

**Table 5:** Cronbach’s alpha if item deleted: assessment of internal consistency and degree of reliability.

Item Assessed (Domain Indicator)	Alfa de Cronbach if Item Deleted	Degree of Consistency
1: Selection of the appropriate cervical collar	0.871	Very good
2: Handling (manipulation) of the cervical collar	0.804	Very good
3: Positioning of the neck in a neutral position	0.635	Moderate
4: Appropriate positioning of healthcare personnel	0.903	Excellent
5: Neck measurement	0.743	Acceptable
6: Correct application of the cervical collar	0.733	Acceptable
7: Bimanual immobilisation	0.661	Moderate
8: Verification of correct placement and comfort	0.808	Very good

Values reflect the overall Cronbach’s alpha that would result if the item were removed from the scale. Values above the overall alpha (α = 0.753) indicate that removing the item would increase the internal consistency of the instrument.

**Table 6:** Component matrix.

Item Assessed (Domain Indicator)	1	2
1: Selection of the appropriate cervical collar	0.29	0.48
2: Handling (manipulation) of the cervical collar	0.33	0.62
3: Positioning of the neck in a neutral position	0.76	0.06
4: Appropriate positioning of healthcare personnel	0.71	0.03
5: Neck measurement	0.13	0.79
6: Correct application of the cervical collar	0.19	0.78
7: Bimanual immobilisation	0.85	0.13
8: Verification of correct placement and comfort	0.42	0.32

Factor loadings ≥ 0.30 are shown. Item assignment to each factor was based on a primary loading ≥ 0.40 and conceptual coherence.

## Data Availability

The dataset is available on reasonable request from the corresponding author due to ethical restrictions.

## Public Involvement Statement

No public involvement in any aspect of this research.

## Guidelines and Standards Statement

This manuscript was prepared following the Reporting Guidelines for Health Care Simulation Research: Extensions to the STROBE Statement ([26]) for observational simulation-based research.

## Use of Artificial Intelligence

During the preparation of this manuscript, the authors used artificial intelligence (AI)-assisted tools (ChatGPT, version GPT-4.0) for the creation of Figure 1 and to support the writing and translation of the Results section. These tools were used exclusively to assist with language expression and presentation of the information and were not involved in any aspect of data processing, analysis, or interpretation. All AI-generated content was carefully reviewed and edited by the authors, who take full responsibility for the final content of the manuscript.
